# Applications and mechanisms of biochar-mycorrhizal synergies in agriculture based on systematic review

**DOI:** 10.7717/peerj.21336

**Published:** 2026-06-04

**Authors:** Hendrix Y. Setyawan, Endrika Widyastuti, Yusron Sugiarto, Nimas Mayang Sabrina Sunyoto, Devy Ulandari, Juwita Dewi, Annisa U. Choirun, Muhammad Arwani, Chiemeka Okoye

**Affiliations:** 1 Department of Agroindustrial Technology, Brawijaya University, Malang, East Java, Indonesia; 2 Department of Biotechnology, Faculty of Agricultural Technology, Brawijaya University, Malang, East Java, Indonesia; 3 Department of Biosystems Engineering, Faculty of Agricultural Technology, Brawijaya University, Malang, East Java, Indonesia; 4 Postgraduate School, Environmental Sciences, Universitas Brawijaya, Malang, Indonesia; 5 Department of Food Engineering, Politeknik Negeri Jember, Jember, Indonesia; 6 Agroindustrial Technology, Universitas Nahdlatul Ulama Indonesia, Jakarta, Indonesia; 7 Centre for Energy, The University of Western Australia, Perth, WA, Australia

**Keywords:** Biochar, Soil health, Sustainable agriculture, Nutrient cycling, Arbuscular mycorrhizal fungi (AMF)

## Abstract

Biochar (BC) and arbuscular mycorrhizal fungi (AMF) have emerged as powerful tools for sustainable agriculture, offering significant benefits for soil health, crop productivity, and ecological resilience. Their combined application has shown synergistic effects; however, key gaps in our understanding remain. This systematic review synthesizes findings from recent studies (*n* = 72) published up to 2025 to examine the synergistic effects of biochar and AMF. A comprehensive search across Scopus, Web of Science, and PubMed employed database-specific Boolean operators combining keywords such as “biochar,” “mycorrhizal fungi,” “soil health,” and “nutrient cycling.” This review highlights the role of biochar in improving soil structure, nutrient availability, and microbial diversity, thereby providing favourable habitats for mycorrhizal colonization. Approximately 78% of reviewed studies reported yield or nutrient-uptake improvements ranging between 15–35% under combined BC-AMF treatments, particularly under environmental stresses like drought or salinity. Synergistic interactions also promoted microbiome shifts, notably increasing the dominance of Glomus and Rhizophagus, which were associated with biochar porosity and nutrient composition. However, uncertainties remain regarding the long-term sustainability of these effects, and the influence of biochar properties on AMF functionality. To fully realize the potential of BC-AMF synergies, future research must focus on standardizing biochar production, exploring molecular and soil–plant–microbe mechanisms, and conducting long-term studies. Collaboration among stakeholders is essential to ensure the scalability and adoption of these technologies, positioning BC–AMF systems as ecologically sustainable alternatives that can support resilient agricultural production while reducing dependence on conventional chemical inputs.

## Introduction

The increasing demand for sustainable agricultural practices has prompted the extensive exploration of innovative soil amendments that enhance productivity while maintaining ecological integrity. Among the various solutions, biochar (BC) and arbuscular mycorrhizal fungi (AMF) have emerged as transformative tools with significant potential to improve soil health, promote plant growth, and enhance the overall agricultural productivity. These nature-based approaches are increasingly viewed as ecologically sustainable alternatives to input-intensive agricultural systems because they can improve nutrient-use efficiency, reduce nutrient losses, and support long-term soil functioning (Woolf et al., 2010; Solaiman, Abbott & Varma, 2014; Shoudho et al., 2024). These amendments not only address critical soil limitations, but also contribute to long-term environmental sustainability by sequestering carbon and improving nutrient-use efficiency, thereby reducing dependence on chemical fertilizers (Hossain et al., 2020; Li et al., 2024). The synergistic application of these technologies in agroecosystems represents a promising avenue for addressing global challenges such as soil degradation, nutrient depletion, and climate change.

Biochar, a carbon-rich material produced through pyrolysis of organic biomass (Setyawan et al., 2024; Sopandi et al., 2025), has been extensively studied for its ability to improve soil properties and fertility. Its high porosity and large surface area enable efficient nutrient retention, thereby reducing leaching and enhancing nutrient availability to plants. For example, studies have demonstrated that biochar effectively retains nitrogen and phosphorus, thereby contributing to higher crop yields (Ahmed, Kurian & Raghavan, 2016; Hossain et al., 2020). Additionally, biochar has shown remarkable potential for alleviating metal toxicity in contaminated soils, supporting microbial diversity and activity, and improving overall soil health (Azadi & Raiesi, 2021; Ibrahim, El-Sherbini & Selim, 2023). These benefits are particularly pronounced in degraded or challenging soils such as those affected by salinity or low organic matter content (Sun et al., 2019; Zhang et al., 2022).

Mycorrhizal fungi form symbiotic relationships with plant roots and play an essential role in nutrient uptake, especially phosphorus uptake, which is often limited to agricultural soils. Through their extensive hyphal networks, these fungi expand the functional surface area of the root system, facilitating access to nutrients that would otherwise remain unavailable to the plants (Zhang et al., 2011; Mickan et al., 2016; Solaiman, Abbott & Varma, 2014). In addition to nutrient acquisition, mycorrhizal associations improve plant resilience to abiotic stresses, such as drought and salinity, by enhancing water uptake and promoting root health (Jabborova et al., 2022; Abed, 2022; Putra, Putra & Widada, 2020). These capabilities position mycorrhizal fungi as critical components of sustainable agriculture, particularly in nutrient-poor or stress environments (Jackson, 2022; Sobhani et al., 2022).

The combination of biochar and mycorrhizal fungi has shown promising results in the enhancing soil health and crop productivity (Rosenthal & Gunn, 2025). For instance, their synergistic effects have been documented in various crop systems, including wheat and flax, where improvements in growth, yield, and water-use efficiency have been observed (Warnock et al., 2010; Rosnina, 2022). In reclaimed soils, biochar supports vegetation establishment by improving soil structure and nutrient availability, which is further augmented by mycorrhizal inoculation (Khdir & Rahman, 2024; Boldt-Burisch et al., 2023). The co-application of these amendments has also enhanced enzymatic activity and nutrient cycling in crops such as corn and spinach, highlighting their potential to transform agroecosystems (Mickan et al., 2016; Carvalho et al., 2016).

Despite substantial evidence supporting the benefits of biochar and mycorrhizal fungi, significant knowledge gaps remain regarding their combined use. Understanding the optimal biochar properties, application rates, and environmental conditions that maximize mycorrhizal colonization is crucial (Bednik, Medynska-Juraszek & Cwielag-Piasecka, 2022; Meng, Cheng & Li, 2024). Furthermore, the mechanisms driving these interactions, particularly the influence of biochar on mycorrhizal activity, require further investigation to inform practical agricultural strategies (Chen, 2023; Neuberger et al., 2024; Idbella et al., 2024). Addressing these gaps will enable the development of targeted applications that optimize the benefits of both amendments in diverse agroecological contexts.

The physical and chemical properties of biochar play pivotal roles in its interaction with mycorrhizal fungi. Biochars with high porosity can serve as refugia for fungal spores and hyphae, enhancing their colonization of plant roots (Chen, 2023; Neuberger et al., 2024). Additionally, the nutrient composition of biochar can directly affect fungal growth and function, with specific types of biochar promoting higher colonization rates and better plant outcomes (Jabborova et al., 2022; Abed, 2022). Understanding these interactions at the mechanistic level is essential for tailoring biochar applications to support mycorrhizal activity and optimize plant health and productivity (Wathira, Peter & Shelia, 2016; Neuberger et al., 2024).

Globally, biochar adoption has expanded rapidly, with significant contributions from Asia, Europe, and North America (Li et al., 2024; Shoudho et al., 2024). This geographical spread underscores the role of biochar as a climate-smart and regionally adaptable solution to soil restoration and sustainable food production.

This review systematically evaluates the synergy between biochar and AMF in modern agriculture. This review aims to synthesize current knowledge, elucidate the underlying mechanisms, identify geographical and methodological gaps, and outline future directions for research and field applications. The overarching goal is to advance the integration of BC–AMF systems into global sustainable agricultural frameworks.

## Methodology

### Search strategy

**Figure 1:**
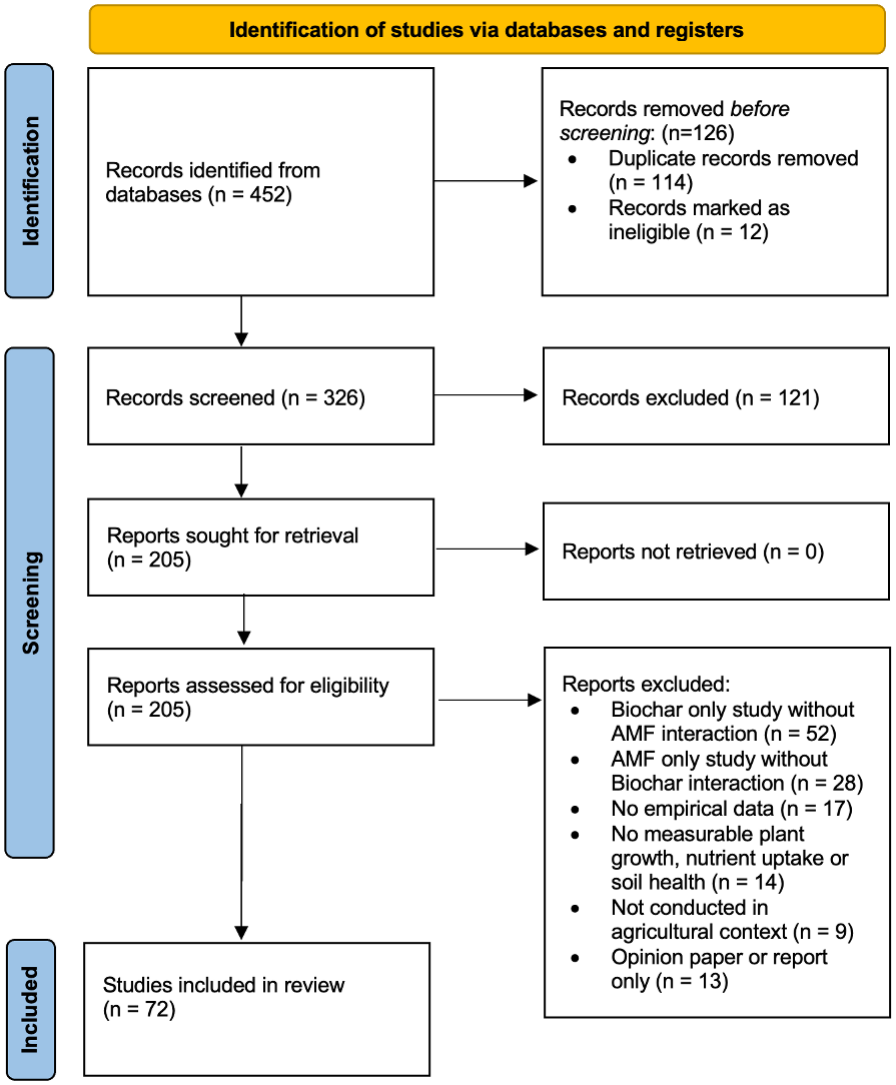
PRISMA flow diagram.

To identify relevant studies on the interactions between biochar and mycorrhizal fungi in agricultural contexts, a comprehensive search was conducted using scientific databases including Scopus, Web of Science, and PubMed. The following keywords and Boolean operators were employed: Keywords: “biochar,” “mycorrhizal fungi,” “arbuscular mycorrhiza,” “plant growth,” “nutrient uptake,” “soil amendment,” “synergistic effects,” “soil health,” and “carbon sequestration.” Search String Example: (“biochar” AND (“mycorrhizal fungi” OR “arbuscular mycorrhiza”) AND (“plant growth” OR “nutrient uptake”) AND (“soil amendment” OR “synergistic effects”) AND (“soil health” OR “carbon sequestration”)). This strategy ensures comprehensive coverage of peer-reviewed articles, conference proceedings, and review papers published until December 2025. Figure 1 shows the PRISMA Flow Diagram illustrating the literature search, identification, screening, and assessment of eligibility, leading to the final set of included studies.

### Inclusion and exclusion criteria

To maintain focus on the synergistic effects of BC and AMF, the following inclusion and exclusion criteria were applied: The inclusion criteria were studies investigating the combined application of biochar and mycorrhizal fungi; research reporting plant growth metrics, nutrient uptake, or soil health indicators; and studies employing experimental designs with control groups, enabling direct comparisons between biochar-mycorrhiza treatments and standalone applications.

The exclusion criteria were studies focusing solely on biochar or AMF without exploring their interactions, articles lacking empirical data or relying solely on anecdotal evidence, and studies that did not include measurable metrics for plant performance or soil health (*e.g.*, qualitative studies). Table 1 summarizes the main characteristics of the included studies, including biochar feedstock, mycorrhizal species, experimental design, and key outcomes.

### Screening and selection process

The screening process involved three sequential phases: (1) title and abstract screening to remove irrelevant or duplicate entries; (2) full-text assessment for methodological soundness and relevance; and (3) final inclusion based on empirical robustness. After screening 452 initial records, 72 studies were included in the final synthesis (Fig. 1). A total of 126 records were removed during the initial filtering stage, consisting of duplicate and ineligible records, before title and abstract screening was conducted on the remaining records. The selection flow was independently verified by two reviewers to ensure reliability and reproducibility.

### Quality assessment

The quality of each included study was evaluated using the Cochrane Risk of Bias tool and the Newcastle-Ottawa Scale (NOS), focusing on the clarity of design, sample size adequacy, data reporting, and reproducibility. Studies were graded as high-, moderate-, or low-quality, and only those meeting the minimum methodological standards were used for synthesis. Overall, most included studies were categorized as moderate to high quality and were therefore considered sufficiently robust for qualitative synthesis.

**Table 1:** Characteristics of included studies.

**Biochar properties**	**Mycorrhiza/Other fungi species**	**Study design**	**Key findings**	**References**
Derived from woody biomass; high carbon content, high porosity	*Glomus mosseae*	Greenhouse experiment; factorial design examining biochar and AMF co-inoculation in sandy soil	Enhanced root colonization, increased phosphorus uptake, improved drought resilience, and higher yield	Jabborova et al. (2022) and Abed (2022)
Produced from poultry litter; nutrient-rich biochar	Indigenous AMF species	Greenhouse pot experiment comparing biochar-amended soils with control	Improved maize biomass and nutrient uptake, particularly under phosphorus-limited conditions	Videgain et al. (2021) and Ghanbari et al. (2019)
Low-temperature pyrolysis biochar with high volatile content	*Rhizophagus irregularis*	Field study on rice with biochar and AMF co-application	Increased yield and grain quality; better soil structure and nutrient retention	Meng, Cheng & Li (2024) and Ma et al. (2023)
Biochar from dry olive residue; high ash content	Various AMF species from contaminated soils	Field study assessing biochar and AMF effects in metal-contaminated soils	Increased microbial diversity, reduced heavy metal bioavailability, enhanced soil health	Siles et al. (2022) and Ibrahim, El-Sherbini & Selim (2023)
Biochar from forestry residues; high surface area	*Glomus fasciculatum*, *Glomus aggregatum*	Controlled experiment examining effects on wheat under salinity stress	Improved wheat growth, water use efficiency, and salt stress tolerance	Solaiman et al. (2010) and Hammer et al. (2015)
High-temperature biochar with greater stability	*Rhizophagus clarus*	Long-term study evaluating biochar and AMF in maize fields	Enhanced nitrogen cycling gene activity, better soil carbon storage, and increased maize yields	Liang et al. (2023) and Xu et al. (2020)
Combined biochar-compost mixture	*Funneliformis mosseae*	Soybean study using biochar, AMF, and compost against *Sclerotinia sclerotiorum* infection	Reduced pathogen incidence, improved soybean yield and nutrient status	Asadabadi, Hage-Ahmed & Steinkellner (2021) and Wathira, Peter & Shelia (2016)
Biochar enriched with pistachio husk nutrients	*Glomus mosseae*	Pot experiment examining biochar-AMF impact on mungbean under heavy metal stress	Reduced nickel distribution in plant tissues; enhanced antioxidant activity and soil enzyme levels	Turan (2021) and Azadi & Raiesi (2021)

### Data analysis

Data were synthesized using a systematic and thematic approach to identify key trends and patterns in the application of BC–AMF. The subthemes included the influence of biochar on soil microbial communities and nutrient dynamics, the effects of biochar-mycorrhizal interactions on plant growth and stress tolerance, and specific applications in diverse soil and environmental conditions. Where quantitative data were available, the results were compared using descriptive statistics to estimate the mean yield or nutrient uptake increases (% ±SD). In addition, summary information from the selected studies was organized into tables to support comparison of biochar feedstock, AMF taxa, experimental conditions, and major agronomic or soil responses across studies.

## Theoretical Framework

This theoretical framework was included to provide a structured conceptual basis for interpreting the mechanisms underlying biochar–mycorrhizal interactions and to guide the synthesis of the evidence presented in the Results and Discussion sections. It integrates physicochemical, biological, and ecological perspectives to explain how biochar properties interact with mycorrhizal fungi and plant systems to influence soil functions and crop performance.

### Biochar properties: influence of feedstock and production methods

Biochar, a carbon-rich material derived from pyrolyzed biomass, has emerged as a key soil amendment owing to its capacity to improve soil health. However, its properties, and consequently its effectiveness, are strongly influenced by the feedstock and production conditions.

Agricultural residues, forestry waste, and animal manure are among the common feedstocks used in biochar production (Setyawan et al., 2024). Each feedstock type yields biochar with unique physical and chemical characteristics. For instance, woody biomass produces biochar with higher carbon content and greater porosity, enhancing its nutrient adsorption and water retention properties (Woolf et al., 2010). Conversely, herbaceous feedstocks often yield biochar with a higher ash content, which can influence the soil pH and nutrient availability. These variations are critical because they determine the interaction between biochar and soil microorganisms, particularly arbuscular mycorrhizal fungi (AMF).

The pyrolysis temperature significantly affects the biochar properties. Higher temperatures (*e.g.*, above 500 °C) typically result in biochar with increased stability, porosity, and surface area, enhancing its long-term efficacy in soils (Khdir & Rahman, 2024). However, lower temperatures retain more volatile compounds, which may enhance short-term nutrient availability and reduce the structural stability of biochar (Shirzad et al., 2024). These temperature-dependent properties influence microbial colonization patterns and nutrient exchange processes at the soil–plant interface.

The interaction between feedstock and pyrolysis conditions underscores the necessity of tailoring biochar production methods to specific agricultural goals such as improving soil structure, nutrient cycling, and crop resilience. Within this theoretical framework, biochar functions as a physicochemical modifier that regulates the habitat suitability for soil microbes and mycorrhizal symbiosis.

### Mycorrhizal fungi: key ecological roles in soil systems

Mycorrhizal fungi are pivotal for maintaining soil health and fostering plant productivity. They form symbiotic relationships with the plant roots and extend their functional capacity through hyphal networks. Mycorrhizal fungi enhance nutrient acquisition, particularly that of phosphorus, by accessing nutrient pools that are otherwise unavailable to plants. This capacity is particularly critical in nutrient-depleted or phosphorus-limited soils (Solaiman, Abbott & Varma, 2014; Zhang et al., 2011).

Beyond nutrient acquisition, mycorrhizal associations bolster plant tolerance to abiotic stresses such as drought and salinity by improving water absorption and strengthening root systems (Putra, Putra & Widada, 2020). Mycorrhizal fungi enhance aeration and water infiltration by promoting soil aggregation. They also foster a diverse and active rhizosphere microbiome that aids in nutrient cycling, suppresses pathogens, and enhances soil health (Kovalchuk et al., 2015; Oviatt, 2020). In addition, AMF can immobilize or sequester heavy metals in their biomass, thereby reducing their bioavailability and toxicity to plants (Ibrahim, El-Sherbini & Selim, 2023). Within this framework, AMF are conceptualized as biological amplifiers (Chen et al., 2025), that translate improvements in soil physicochemical conditions (Fišarová et al., 2024) into enhance plant nutrient uptake and stress resistance.

### Potential synergies between biochar and mycorrhizal fungi

The integration of biochar and mycorrhizal fungi into soil systems offers synergistic benefits that can amplify their individual effects on soil health and plant productivity (Barna et al., 2020). The porous structure of biochar provides a microhabitat that protects mycorrhizal fungi from environmental stressors such as predation and desiccation, facilitating greater fungal colonization rates (Giono et al., 2021; Neuberger et al., 2024). Biochar improves the soil pH, moisture retention, and nutrient availability, thereby creating an environment conducive to mycorrhizal activity. This enhancement in turn leads to improved nutrient uptake and plant growth (Sobhani et al., 2022). Studies have indicated that the combination of biochar and mycorrhizal inoculation improves plant growth, nutrient use efficiency, and resilience to abiotic stresses compared with their standalone applications (Wathira, Peter & Shelia, 2016; Wurzburger & Brookshire, 2017). Notably, shifts in AMF community composition, including the increased dominance of Glomus and Rhizophagus species, have been linked to biochar porosity and nutrient characteristics (Neuberger et al., 2024).

This synergy can be theoretically framed as a soil–microbe–plant feedback system, in which biochar modifies the physical and chemical soil environment, AMF enhance biological nutrient transfer, and plants respond through improved growth and stress resilience.

### Implications for sustainable agriculture

The symbiosis between biochar and mycorrhizal fungi highlights a pathway to achieving sustainable agricultural goals. By integrating carbon sequestration, improved nutrient-use efficiency, and biological soil enhancement, this synergy aligns with global priorities to reduce chemical fertilizer dependency and increase resilience to climate variability. In this sense, BC–AMF systems may be viewed as ecologically sustainable management strategies that support productive agriculture while reinforcing long-term soil health. Within the context of this review, the theoretical framework serves as a bridge between empirical findings and sustainability outcomes, supporting the evaluation of biochar–AMF systems as nature-based solutions for long-term soil and crop management (Reddy, 2024; Sousa et al., 2024).

## Results and Discussion

Overall, the 72 studies included in this review showed a predominance of greenhouse and pot-based experiments, with fewer long-term field studies, indicating that current evidence is stronger for short-term mechanistic understanding than for large-scale field validation. As summarized in Tables 1–4, the reviewed studies commonly used biochar derived from woody biomass, crop residues, and manure-based feedstocks, while Glomus and Rhizophagus were the most frequently reported AMF taxa. Across the dataset, the most consistent outcomes of BC–AMF co-application were improvements in microbial activity, nutrient uptake, plant biomass, and yield, particularly under drought, salinity, nutrient-poor, or contaminated soil conditions. These trends provide the basis for the thematic discussion below.

**Table 2:** Biochar-mycorrhiza studies.

**Crop**	**Soil type**	**Biochar type**	**Mycorrhiza species**	**Key outcomes**	**References**
Soybean	Sandy soil	Woody biochar; high porosity	*Glomus mosseae*	Improved drought tolerance, increased nitrogen and phosphorus uptake	Jabborova et al. (2022) and Abed (2022)
Wheat	Saline soil	High-temperature biochar; nutrient-rich	*Glomus fasciculatum*, *Glomus aggregatum*	Enhanced growth, water use efficiency, and salt stress resilience	Solaiman et al. (2010) and Hammer et al. (2015)
Cowpea	Metal-contaminated soil	Olive residue-based biochar	Indigenous AMF species	Reduced heavy metal bioavailability, increased microbial diversity	Ibrahim, El-Sherbini & Selim (2023) and Siles et al. (2022)
Rice	Low organic matter soil	Low-temperature biochar; high volatile content	*Rhizophagus irregularis*	Improved yield, enhanced nitrogen cycling and potassium availability	Meng, Cheng & Li (2024) and Ma et al. (2023)
Peanut	Sandy loam soil	Agricultural waste biochar; high ash content	*Funneliformis mosseae*	Higher phosphorus and zinc uptake, improved pod yield	Cheng et al. (2021) and Sobhani et al. (2022)
Upland rice	Acidic soil	Biochar enriched with organic nutrients	*Glomus mosseae*	Increased shoot biomass, higher phosphorus and calcium uptake	Kilowasid et al. (2023) and Rakian (2024)
Potato	Nutrient-depleted soil	Wood biochar; high stability	*Rhizophagus clarus*	Increased tuber yield and nitrogen use efficiency	Liu et al. (2016) and Jackson (2022)
Mung bean	Sandy soil with arsenic	Selenium-rich biochar	*Glomus mosseae*	Reduced arsenic uptake, increased selenium bioavailability, higher antioxidant activity	Alam et al. (2019) and Turan (2021)

**Table 3:** Biochar-mycorrhiza synergy outcomes.

**Soil type**	**Crop**	**Nutrient uptake metrics**	**Yield improvements**	**References**
Sandy soil	Soybean	Increased phosphorus and nitrogen uptake	Enhanced biomass under drought conditions	Jabborova et al. (2022) and Abed (2022)
Saline soil	Wheat	Higher nitrogen and phosphorus uptake	Improved grain yield and water use efficiency	Solaiman et al. (2010) and Hammer et al. (2015)
Metal-contaminated soil	Cowpea	Reduced heavy metal uptake; improved phosphorus availability	Increased root and shoot biomass	Ibrahim, El-Sherbini & Selim (2023) and Siles et al. (2022)
Calcareous soil	Wheat	Enhanced phosphorus and potassium availability	Increased yield and root colonization	Khdir & Rahman (2024) and Zhang et al. (2011)
Low organic matter soil	Rice	Enhanced potassium uptake; improved nitrogen cycling	Increased grain yield and amino acid content	Meng, Cheng & Li (2024) and Ma et al. (2023)
Sandy loam soil	Peanut	Improved phosphorus and zinc uptake	Higher pod yield and nutrient use efficiency	Sobhani et al. (2022) and Cheng et al. (2021)
Acidic soil	Upland rice	Higher phosphorus and calcium uptake	Increased shoot biomass and tolerance to acid stress	Kilowasid et al. (2023) and Rakian (2024)
Nutrient-depleted soil	Potato	Enhanced nitrogen, phosphorus, and potassium uptake	Improved tuber yield and quality	Liu et al. (2016) and Jackson (2022)
Sandy soil with arsenic	Mung bean	Increased selenium and reduced arsenic uptake	Higher chlorophyll content and antioxidant enzyme activity	Alam et al. (2019) and Turan (2021)

**Table 4:** Comparison of biochar-only, mycorrhiza-only, and combined applications on crop yield and soil properties.

**Treatment**	**Crop**	**Soil properties**	**Crop yield**	**References**
Biochar-Only	Wheat	Improved soil pH and nutrient retention	Moderate increase in grain yield under saline conditions	Solaiman et al. (2010) and Hammer et al. (2015)
Mycorrhiza-Only	Wheat	Enhanced phosphorus availability and microbial activity	Increased root biomass and water use efficiency	Solaiman et al. (2010) and Hammer et al. (2015)
Combined Application	Wheat	Synergistic improvements in pH, nutrient cycling, and soil structure	Significant increase in grain yield and stress tolerance	Solaiman et al. (2010) and Hammer et al. (2015)
Biochar-Only	Rice	Increased soil carbon and potassium availability	Higher yield under nutrient-depleted conditions	Ma et al. (2023) and Meng, Cheng & Li (2024)
Mycorrhiza-Only	Rice	Enhanced nitrogen and phosphorus cycling	Moderate increase in grain yield	Meng, Cheng & Li (2024) and Ma et al. (2023)
Combined Application	Rice	Improved soil enzymatic activity, carbon storage, and nutrient retention	Substantial increase in yield and grain quality	Ma et al. (2023) and Meng, Cheng & Li (2024)
Biochar-Only	Soybean	Improved soil moisture retention	Slight increase in biomass	Abed (2022) and Jabborova et al. (2022)
Mycorrhiza-Only	Soybean	Increased phosphorus uptake and root colonization	Moderate biomass improvement	Abed (2022) and Jabborova et al. (2022)
Combined Application	Soybean	Enhanced nutrient uptake, soil microbial diversity, and drought resilience	Significant increase in biomass under drought stress	Jabborova et al. (2022) and Abed (2022)
Biochar-Only	Cowpea	Reduced heavy metal availability	Improved root growth in metal-contaminated soil	Ibrahim, El-Sherbini & Selim (2023) and Siles et al. (2022)
Mycorrhiza-Only	Cowpea	Increased soil microbial diversity	Slight increase in shoot biomass	Ibrahim, El-Sherbini & Selim (2023) and Siles et al. (2022)
Combined Application	Cowpea	Synergistic effects on metal immobilization and microbial activity	Substantial increase in biomass and nutrient use efficiency	Ibrahim, El-Sherbini & Selim (2023) and Siles et al. (2022)
Biochar-Only	Peanut	Enhanced phosphorus retention	Increased pod yield under sandy soil conditions	Cheng et al. (2021) and Sobhani et al. (2022)
Mycorrhiza-Only	Peanut	Improved phosphorus solubilization	Slight increase in pod yield	Cheng et al. (2021) and Sobhani et al. (2022)
Combined Application	Peanut	Improved nutrient uptake, enzymatic activity, and soil aggregation	Significant pod yield increase	Cheng et al. (2021) and Sobhani et al. (2022)

### Impact of biochar on microbial diversity in agricultural soils

The ability of biochar to enhance microbial diversity and activity in agricultural soils has been consistently demonstrated. Across the reviewed studies, positive responses of microbial abundance, activity, or community structure were among the most frequently reported outcomes, especially in experiments combining biochar with AMF inoculation (Tables 2 and 3). Its highly porous structure and substantial surface area create a conducive environment for colonization by beneficial microorganisms. This habitat supports a wide range of microbes, including plant growth-promoting rhizobacteria (PGPR) and mycorrhizal fungi, which play vital roles in nutrient cycling and soil fertility (Xu et al., 2020; Solaiman, Abbott & Murphy, 2019).

Biochar improves nutrient availability and microbial diversity by modifying soil pH, increasing cation exchange capacity, and reducing nutrient leaching. For example, biochar increases phosphorus availability and stimulates the growth of phosphorus-solubilizing microbes and mycorrhizal fungi (Herawati et al., 2021; Li & Cai, 2021; Wen et al., 2024).

Biochar can also improve the soil structure and microbial resilience. The enhanced soil structure due to biochar addition supports diverse microbial communities by improving aeration and water retention. These changes contribute to higher microbial enzymatic activity, thereby boosting soil health and crop productivity (Kilowasid et al., 2023; Brantley et al., 2016). The relationship between biochar and soil microbes underscores the potential of biochar as a tool to promote microbial diversity, which is essential for maintaining productive and resilient agricultural ecosystems. Most of the reviewed studies reported positive microbial responses to biochar application, particularly when it was combined with AMF inoculation (Tables 2 and 3).

### Mechanisms of biochar-enhanced mycorrhizal colonization

The ability of biochar to improve mycorrhizal colonization stems from its multiple interacting mechanisms. Biochar enhances soil porosity and provides attachment sites for mycorrhizal hyphae, facilitating the fungal colonization of plant roots. This structural improvement creates a supportive environment for fungal proliferation (Meier et al., 2015; Asadabadi, Hage-Ahmed & Steinkellner, 2021). The influence of biochar on soil pH and nutrient availability significantly affects mycorrhizal activity (Ndiate et al., 2021). Biochar creates a more hospitable environment for fungi by increasing the soil pH in acidic environments. Additionally, its ability to retain and supply critical nutrients such as phosphorus further stimulates fungal colonization (Turan, 2021; McCormack et al., 2019). The evidence summarized in Tables 2 and 4 indicates that colonization responses were generally more favourable under biochar with high porosity and moderate nutrient levels, whereas excessively nutrient-rich biochar could suppress colonization. Several studies summarized in Table 2 indicate that biochars with high porosity and moderate nutrient content are associated with higher AMF root colonization rates, particularly for Glomus and Rhizophagus species (Neuberger et al., 2024). The presence of biochar can modify root exudates, thereby enhancing the signalling pathways that promote mycorrhizal symbiosis. This interaction strengthens the symbiotic relationship, leading to an improved nutrient exchange between fungi and plants (Jatuwong et al., 2024; Neuberger et al., 2024).

### Molecular pathways activated during biochar-mycorrhizal interactions

One critical aspect of biochar-mycorrhizal interactions is the activation of specific molecular pathways that facilitate symbiosis and enhance plant resilience to stress (Hashem et al., 2019). Studies have demonstrated that biochar can influence the expression of genes related to symbiosis and nutrient uptake in plants. For example, Suetsugu et al. (2017) revealed that specific symbiosis-related genes in mycorrhizal orchid roots were upregulated, particularly in mycoheterotrophy variants, thereby emphasizing the role of mycorrhizal fungi in nutrient acquisition. Although the present study focused on orchids, its implications extend to other plants, suggesting that biochar may promote similar gene expression in various mycorrhizal systems.

The Jasmonic Acid (JA) and ethylene (ET) signalling pathways are particularly relevant in mycorrhizal plants under stress conditions. These pathways play essential roles in plant responses to biotic and abiotic stressors. Rashad et al. (2021) demonstrated that AMF colonization induced the overexpression of stress-responsive genes, such as JERF3, enhancing tolerance to combined biotic and abiotic stresses. By supporting AMF establishment, biochar indirectly contributes to the activation of defence-related pathways, as reported in several stress-related studies (Table 3).

In addition to stress-related signalling, biochar–AMF interactions influence plant respiratory and metabolic pathways. Liu et al. (2014) showed that AMF-colonized rice plants under low-temperature stress exhibited improved respiratory efficiency owing to the modulation of the alternative oxidase (AOX) and cytochrome c oxidase (COX) pathways. These physiological responses align with the improved growth and yield outcomes summarized in the rice-based studies (Tables 3 and 4).

### Effects of biochar on mycorrhizal gene expression related to stress resilience

The ability of biochar to enhance mycorrhizal colonization extends to its influence on the expression of stress-resilient genes in plants. Campos-Soriano, García-Martínez & Segundo (2011) demonstrated that mycorrhizal symbiosis induces systemic activation of defence-related genes in rice, providing resistance against pathogens. When biochar is applied, it can amplify this systemic response by improving soil structure, nutrient availability, and microbial habitat. These enhancements create an optimal environment for mycorrhizal fungi, which in turn strengthens the plant defence mechanisms.

Another critical aspect of biochar-mycorrhizal interactions is the modulation of genes involved in nutrient transport. Phosphate transporters, which are crucial for phosphorus acquisition in nutrient-poor soils, are significantly affected by mycorrhizal symbiosis. Solaiman, Abbott & Murphy (2019) highlighted the modulation of these transporters in mycorrhizal plants and demonstrated the potential of biochar to support nutrient uptake under challenging soil conditions. By enhancing the bioavailability of essential nutrients, biochar fosters mycorrhizal colonization and improves the ability of plants to cope with nutrient stress.

The systemic benefits of biochar can extend beyond nutrient transport. Rashad et al. (2021) noted that biochar application could improve the resilience of mycorrhizal plants by promoting the expression of genes associated with oxidative stress management. This includes upregulation of antioxidant enzyme pathways, which mitigate the damaging effects of reactive oxygen species under stressful conditions. Overall, these findings suggest that the contribution of BC–AMF systems to stress resilience is not only structural and nutritional, but also physiological and transcriptional, although this evidence is still based on a limited subset of studies. These interactions highlight the potential of biochar to enhance the functional role of mycorrhizal fungi in plant stress response.

### Biochar’s influence on nutrient transport and stress management

The interaction between biochar and mycorrhizal fungi significantly affects nutrient transport mechanisms, particularly in phosphorus-limited soils. Mycorrhizal plants rely on extensive fungal hyphal networks to access nutrients. Biochar enhances these interactions by altering the physical and chemical properties of the soil, thereby improving the availability of essential nutrients, such as phosphorus, potassium, and nitrogen (Solaiman, Abbott & Murphy, 2019). This effect is particularly pronounced in nutrient-depleted or marginal soils, where traditional fertilization methods are less effective.

The influence of biochar on stress-responsive genes is a critical research area. By enhancing soil water retention and buffering pH, biochar mitigates environmental stressors and creates a favourable microenvironment for mycorrhizal fungi. This improved habitat supports the fungal networks that activate systemic stress responses in plants. For example, Liu et al. (2014) found that AMF-colonized rice plants with biochar amendments exhibited increased expression of genes involved in oxidative stress tolerance, highlighting the role of biochar in supporting plant health under abiotic stress conditions. As reflected in Tables 3 and 4, these mechanisms were commonly associated with improved nutrient uptake, biomass accumulation, and greater stability of plant performance under stress.

### Synergistic applications of biochar and mycorrhiza in enhancing crop yields

Empirical evidence strongly supports the synergistic effects of combined application of biochar and AMF on crop productivity. Across diverse crops and soil types, co-application consistently results in higher biomass production, improved nutrient uptake, and enhanced stress tolerance compared with standalone treatments (Liu et al., 2016; Jabborova et al., 2022).

As summarized in Table 3, most of the reviewed studies reported increases in yield or biomass under combined biochar–AMF treatments, particularly under drought, salinity, or nutrient-limited conditions. The comparative evidence presented in Table 4 further demonstrates that combined applications outperform biochar- or AMF-only treatments in terms of soil quality improvement and yield stability. This general trend is consistent with the overall synthesis, in which approximately 78% of studies reported yield or nutrient-uptake improvements, most commonly within the range of 15–35%. For example, in rice and wheat systems, the application of integrated biochar–AMF improves nutrient availability, enzymatic activity, and grain yield (Ma et al., 2023; Meng, Cheng & Li, 2024; Solaiman et al., 2010). These findings indicate a consistent pattern across agroecosystems, rather than isolated, case-specific effects.

### Tailoring biochar applications for optimal outcomes

Although the benefits of biochar-mycorrhizal interactions are well documented, their outcomes are highly dependent on the specific properties of biochar and the environmental context. For example, the porosity and surface area of biochar directly influence its ability to support mycorrhizal colonization. Mason et al. (2025) emphasized that biochar provides refugia for fungal hyphae, protecting them from grazers and desiccation. However, excessive nutrient concentrations in biochar, particularly phosphorus, can inhibit fungal colonization (Solaiman, Abbott & Murphy, 2019). The evidence summarized in Tables 2 and 4 suggests that biochars with balanced nutrient content and moderate application rates are the most effective in promoting AMF activity and crop performance. Therefore, understanding plant–microbe signalling dynamics is critical for designing biochar formulations that enhance, rather than inhibit, mycorrhizal associations (Kayama et al., 2022).

### Limitations and future directions

Despite these promising results, challenges remain in optimizing biochar-AMF applications. The effectiveness of biochar and mycorrhizal combinations can vary depending on soil type, crop species, and environmental conditions. Most of the reviewed studies are short-term and site-specific, limiting inferences on long-term sustainability and scalability. Further research is required to identify site-specific protocols. Although the short-term benefits are well documented, the long-term implications of these applications on soil health and productivity require further investigation. Tailoring biochar properties (*e.g.*, porosity and nutrient composition) to maximize its compatibility with mycorrhizal fungi and specific crops remains a key area for future research (Khdir & Rahman, 2024; Jackson, 2022).

Economic analyses are critical for evaluating the feasibility of large-scale biochar applications. Although biochar offers numerous agronomic and environmental benefits, its high production costs may limit its adoption. Developing cost-effective biochar production technologies and demonstrating their value through field trials is key to promoting their use in sustainable agriculture. In addition, because the quality assessment indicated that most included studies were of moderate to high quality but predominantly short-term and controlled-environment based, future research should prioritize longer-duration field experiments, clearer reporting standards, and more comparable quantitative outcome measures.

## Conclusion

This review highlights the potential of integrating biochar and mycorrhizal fungi into modern agricultural practices. Biochar, owing to its porous structure and nutrient-retentive properties, creates an enhanced habitat for soil microbes, including mycorrhizal fungi. These fungi amplify nutrient uptake, improve plant resilience to environmental stress, and foster soil health. Together, these amendments synergistically improved the soil structure, nutrient cycling, and plant productivity. Together, BC–AMF systems consistently enhance soil structure, nutrient cycling, and plant productivity across a wide range of crops and soil conditions.

Across the reviewed literature, combined BC–AMF applications more frequently improved crop performance under abiotic stresses, such as drought, salinity, and nutrient limitation, than standalone treatments. Key findings include the ability of biochar to promote microbial diversity and provide a conducive environment for mycorrhizal colonization through physical and chemical soil enhancement. Across the reviewed literature, combined BC–AMF applications more frequently resulted in improved crop performance under abiotic stresses, such as drought, salinity, and nutrient limitation, than standalone treatments.

Key unresolved areas include the precise mechanisms by which biochar enhances mycorrhizal colonization and functionality, variability of outcomes across different biochar types, and long-term effects on soil health and crop productivity. Overall, the available evidence supports BC–AMF systems as promising tools for sustainable agriculture, but their wider application will depend on clearer optimization of biochar properties, crop-specific protocols, and long-term field validation.

##  Supplemental Information

10.7717/peerj.21336/supp-1 Supplemental Information 1 PRISMA checklist.

10.7717/peerj.21336/supp-2 Supplemental Information 2 Intended Audience.

## Additional Information and Declarations

### Data Availability

The following information was supplied regarding data availability: This is a Systematic review/Meta-analysis.
