# Supplementary material for: Applications and mechanisms of biochar-mycorrhizal synergies in agriculture based on systematic review
Source: PeerJ. 2026 Jun 4;14:e21336. doi: 10.7717/peerj.21336 (PMC13242744; doi:10.7717/peerj.21336)
Supplement: Supplemental Information 1 [file peerj-14-21336-s001.docx]

| **Section and Topic** | **Item #** | **Checklist item** | **Location where item is reported** |
| --- | --- | --- | --- |
| **TITLE** | | |  |
| Title | 1 | Identify the report as a systematic review. | Title Page, Abstract: Page1, Line1, Line35-50 |
| **ABSTRACT** | | |  |
| Abstract | 2 | See the PRISMA 2020 for Abstracts checklist. | Abstract: Page1, Line 36-50 |
| **INTRODUCTION** | | |  |
| Rationale | 3 | Describe the rationale for the review in the context of existing knowledge. | Introduction: Page 2, Line 75-92 |
| Objectives | 4 | Provide an explicit statement of the objective(s) or question(s) the review addresses. | End of Introduction: Page 3 Line 118 |
| **METHODS** | | |  |
| Eligibility criteria | 5 | Specify the inclusion and exclusion criteria for the review and how studies were grouped for the syntheses. | Methods: 2.2: Page 6 Line138 |
| Information sources | 6 | Specify all databases, registers, websites, organisations, reference lists and other sources searched or consulted to identify studies. Specify the date when each source was last searched or consulted. | Methods: 2.1: Page 6 Line 126 |
| Search strategy | 7 | Present the full search strategies for all databases, registers and websites, including any filters and limits used. | Methods: 2.1: Page 6 Line 149 |
| Selection process | 8 | Specify the methods used to decide whether a study met the inclusion criteria of the review, including how many reviewers screened each record and each report retrieved, whether they worked independently, and if applicable, details of automation tools used in the process. | Methods: 2.3: Page 6 Line 138 |
| Data collection process | 9 | Specify the methods used to collect data from reports, including how many reviewers collected data from each report, whether they worked independently, any processes for obtaining or confirming data from study investigators, and if applicable, details of automation tools used in the process. | Methods: 2.3, Tables: Page 7 Line 158-161 |
| Data items | 10a | List and define all outcomes for which data were sought. Specify whether all results that were compatible with each outcome domain in each study were sought (e.g. for all measures, time points, analyses), and if not, the methods used to decide which results to collect. | Methods: 2.3: Page 7 Line 162 |
|  | 10b | List and define all other variables for which data were sought (e.g. participant and intervention characteristics, funding sources). Describe any assumptions made about any missing or unclear information. | Methods: 2.3: Page 7 Line 149 |
| Study risk of bias assessment | 11 | Specify the methods used to assess risk of bias in the included studies, including details of the tool(s) used, how many reviewers assessed each study and whether they worked independently, and if applicable, details of automation tools used in the process. | Methods: 2.4: Page 7 Line 162 |
| Effect measures | 12 | Specify for each outcome the effect measure(s) (e.g. risk ratio, mean difference) used in the synthesis or presentation of results. | Methods: 2.4: Page 7 Line 150 |
| Synthesis methods | 13a | Describe the processes used to decide which studies were eligible for each synthesis (e.g. tabulating the study intervention characteristics and comparing against the planned groups for each synthesis (item #5)). | Methods: 2.3: Page 5 Line 149 |
|  | 13b | Describe any methods required to prepare the data for presentation or synthesis, such as handling of missing summary statistics, or data conversions. | 2.3. Screening and Selection Process; 2.5. Data Analysis: Page 6-7 Line 169 |
|  | 13c | Describe any methods used to tabulate or visually display results of individual studies and syntheses. | 2.3. Screening and Selection Process Line 149; |
|  | 13d | Describe any methods used to synthesize results and provide a rationale for the choice(s). If meta-analysis was performed, describe the model(s), method(s) to identify the presence and extent of statistical heterogeneity, and software package(s) used. | Methods: 2.5: Line 169 |
|  | 13e | Describe any methods used to explore possible causes of heterogeneity among study results (e.g. subgroup analysis, meta-regression). | 2.5. Data Analysis; Line 169 |
|  | 13f | Describe any sensitivity analyses conducted to assess robustness of the synthesized results. | Not explicitly described |
| Reporting bias assessment | 14 | Describe any methods used to assess risk of bias due to missing results in a synthesis (arising from reporting biases). | 2.4. Quality Assessment: Page 7 Line 162 |
| Certainty assessment | 15 | Describe any methods used to assess certainty (or confidence) in the body of evidence for an outcome. | 2.4. Quality Assessment: Page 7 Line 162 |
| **RESULTS** | | |  |
| Study selection | 16a | Describe the results of the search and selection process, from the number of records identified in the search to the number of studies included in the review, ideally using a flow diagram. | Fig 1: Page 5 Line 139 |
|  | 16b | Cite studies that might appear to meet the inclusion criteria, but which were excluded, and explain why they were excluded. | 2.2. Inclusion and Exclusion Criteria; 2.3. Screening: Page 7 Linen 149 |
| Study characteristics | 17 | Cite each included study and present its characteristics. | References; 2.2–2.3, Tables: Page 7–8; references pages 18 Line 416 |
| Risk of bias in studies | 18 | Present assessments of risk of bias for each included study. | 2.4. Quality Assessment: Page 7 line 162 |
| Results of individual studies | 19 | For all outcomes, present, for each study: (a) summary statistics for each group (where appropriate) and (b) an effect estimate and its precision (e.g. confidence/credible interval), ideally using structured tables or plots. | Results; Tables: Page 8–12 (Tables 2, 3, 4) Line 246, Line 316 and line 325 |
| Results of syntheses | 20a | For each synthesis, briefly summarise the characteristics and risk of bias among contributing studies. | 4.6 Synergistic Applications Line 314 |
|  | 20b | Present results of all statistical syntheses conducted. If meta-analysis was done, present for each the summary estimate and its precision (e.g. confidence/credible interval) and measures of statistical heterogeneity. If comparing groups, describe the direction of the effect. | Results (throughout); Tables: Page 8-12 Line 228 - 354 |
|  | 20c | Present results of all investigations of possible causes of heterogeneity among study results. | 4.7 Tailoring Applications: Page 11 Line 328 |
|  | 20d | Present results of all sensitivity analyses conducted to assess the robustness of the synthesized results. | Not explicitly described |
| Reporting biases | 21 | Present assessments of risk of bias due to missing results (arising from reporting biases) for each synthesis assessed. | Not explicitly described |
| Certainty of evidence | 22 | Present assessments of certainty (or confidence) in the body of evidence for each outcome assessed. | Not explicitly described |
| **DISCUSSION** | | |  |
| Discussion | 23a | Provide a general interpretation of the results in the context of other evidence. | Result and Discussion: Line 228 |
|  | 23b | Discuss any limitations of the evidence included in the review. | Section 4.8: Page14 Line 342 |
|  | 23c | Discuss any limitations of the review processes used. | 4.8 Limitations and Future Directions Page14 Line 342 |
|  | 23d | Discuss implications of the results for practice, policy, and future research. | 4.8 Limitations and Future Directions: Page14 Line 342 Page 12-13 |
| **OTHER INFORMATION** | | |  |
| Registration and protocol | 24a | Provide registration information for the review, including register name and registration number, or state that the review was not registered. | Not registered |
|  | 24b | Indicate where the review protocol can be accessed, or state that a protocol was not prepared. | Not prepared |
|  | 24c | Describe and explain any amendments to information provided at registration or in the protocol. | Not described |
| Support | 25 | Describe sources of financial or non-financial support for the review, and the role of the funders or sponsors in the review. | Funding Statement: Page16 Line 397 |
| Competing interests | 26 | Declare any competing interests of review authors. | Disclosure of Interest: Page16 Line 397 |
| Availability of data, code and other materials | 27 | Report which of the following are publicly available and where they can be found: template data collection forms; data extracted from included studies; data used for all analyses; analytic code; any other materials used in the review. | Data Availability Statement: Page16 Line 397 |

*From:*  Page MJ, McKenzie JE, Bossuyt PM, Boutron I, Hoffmann TC, Mulrow CD, et al. The PRISMA 2020 statement: an updated guideline for reporting systematic reviews. BMJ 2021;372:n71. doi: 10.1136/bmj.n71. This work is licensed under CC BY 4.0. To view a copy of this license, visit <https://creativecommons.org/licenses/by/4.0/>
