# Supplementary material for: Applications and mechanisms of biochar-mycorrhizal synergies in agriculture based on systematic review
Source: PeerJ. 2026 Jun 4;14:e21336. doi: 10.7717/peerj.21336 (PMC13242744; doi:10.7717/peerj.21336)
Supplement: Supplemental Information 2 [file peerj-14-21336-s002.docx]

**Intended Audience**

The intended audience for this systematic review includes the following key groups:

1. *Academic Researchers and Scientists*

- Especially those in the fields of soil science, agronomy, microbial ecology, plant physiology, and sustainable agriculture.
- The review provides a synthesis of current findings and identifies knowledge gaps, making it valuable for informing future experimental design and interdisciplinary research.

1. *Agricultural Practitioners and Agronomists*

Professionals involved in soil management, crop productivity enhancement, or organic farming can gain insights into how combining biochar and mycorrhizal fungi can improve yield and soil health—particularly under stress conditions like drought, salinity, and metal contamination.

1. *Environmental Scientists and Soil Health Experts*

The review offers mechanisms and empirical evidence relevant to those working on soil restoration, carbon sequestration, and reducing agrochemical dependence, thus aligning with environmental sustainability goals.

1. *Policy Makers and Extension Specialists*

Those involved in creating or advising on agricultural policies, climate-smart agriculture, or resource-efficient technologies will find this synthesis useful for translating scientific insights into practical and scalable interventions.

1. *Graduate Students and Educators*

This work serves as a learning resource for postgraduate students and lecturers seeking a structured overview of biochar–mycorrhiza interactions, making it ideal for use in academic coursework, thesis development, or curriculum design.
